# Impact of Perceived Influence, Virtual Interactivity on Consumer Purchase Intentions Through the Path of Brand Image and Brand Expected Value

**DOI:** 10.3389/fpsyg.2022.947916

**Published:** 2022-07-28

**Authors:** Xinzhong Jia, Abdul Khaliq Alvi, Muhammad Aamir Nadeem, Nadeem Akhtar, Hafiz Muhammad Fakhar Zaman

**Affiliations:** ^1^School of Industry and Commerce, Shandong Management University, Jinan, China; ^2^Department of Management Sciences, Lahore Garrison University, Lahore, Pakistan; ^3^School of Management, Universiti Sains Malaysia, Gelugor, Malaysia; ^4^Department of Management Sciences, Yanbu University College, Yanbu, Saudi Arabia; ^5^School of Management and Economics, Kunming University of Science and Technology, Kunming, China

**Keywords:** perceived influence, virtual interactivity, brand image, brand expected value, consumer purchase intentions, m-commerce

## Abstract

Many researchers are currently showing interest in researching consumers who are purchasing the products with the help of new tools, and new kinds of markets are emerging rapidly. M-commerce is a prevalent mode of marketing and is famous among young people of Pakistan. Current research is planned to check the status of consumer purchase intentions (PIs) using perceived influence, virtual interactivity, brand image, and brand expected value among customers who purchase their products with the help of m-commerce. Data was collected from customers who were engaged in buying with the help of m-commerce by using the convenience sampling technique and 227 complete questionnaires were used in final analysis. This research examines the direct impact of perceived influence, virtual interactivity, brand image, and brand expected value on PIs and finds the indirect effect of brand image and brand expected value on the relationships of perceived influence and virtual interactivity with PIs. Results indicate that all the hypotheses of direct relationships are accepted except the hypothesis for the relation of virtual interactivity with consumer PIs. Virtual interactivity has an insignificant positive impact on consumer PIs. Brand expected value has a strong positive effect on consumer PIs among all. The current study proposed four mediational hypotheses. All the proposed mediational hypotheses are accepted.

## Introduction

Current research explains the relations of proposed variables based on two theories, 1-media dependency theory, and 2-self-congruity theory. Companies are increasingly considering social media, Mobile use as a strategic tool for promoting their products and brands and as well as for building strong customer relationships (Shiau et al., 2018). Practitioners and researchers interested in brand concerns and platforms of social media, including mobile users, have taken notice of this regard (Alalwan et al., 2017). M-commerce and social media use relevant advertising for brands through several active techniques and processes, like influencer marketing, online brand communities, micro blogging, and blogging; in this regard, the company makes necessary arrangements (e.g., Childers and Maggard-Gibbons, 2018; Kapoor et al., 2018). These practices enable companies to participate in shared activities with prospective consumers to share brand information and collaborative practices using influencers to promote their items to their followers (Hajli et al., 2017; Sokolova and Kefi, 2020).

Technological breakthroughs have impacted global societies. Technological advances have a significant impact on society as a whole. Due to the development of new technological approaches and algorithms, access to virtually any type of information is now possible almost instantaneously. As technology has become more widespread in the commercial world, e-commerce has changed during the previous decade. Social commerce, a subcategory of e-commerce, is widely used and preferred by consumers. Mobile commerce has made a significant impact on social interaction. There are around 3.3 billion smartphone users globally (Marriott et al., 2017). Smartphones have a considerable effect on consumption habits due to their widespread use by consumers. Users of cell phones have a significant impact on each other’s lives as they become more interconnected and consumers are influenced by each other.

Due to its enormous impact on business and industry, the rise of mobile commerce (m-commerce) has been widely studied by practitioners and academics alike (Marriott et al., 2017). It is a new form of electronic commerce that does not require a desktop computer and instead relies on mobile devices connected to a wireless telecommunications network to perform transactions (Holmes et al., 2014). Customers have never had it so good when it comes to online purchasing because of this one-of-a-kind feature. Sales of e-commerce in the United States made up 24% of total e-commerce sales in 2016, significantly from 11% in 2012. In the United States, m-commerce expanded roughly three times as fast as e-commerce (Saleh, 2017). By 2020, it is predicted that mobile commerce will account for at least 45% of the whole e-commerce market in the United States, earning $284 billion in annual sales (Intelligence, 2015). Boston Consulting Group (2013) reported that mobile commerce is the driving factor behind the next wave of retail transformation and a new source of competitive advantage for enterprises. Holmes et al. (2014) argued that product-specific m-commerce studies could yield more relevant results while eliminating the confounding effects of product variances. According to recent trends in online buying, customers are spending increasing time and money on mobile retail platforms (Bilgihan et al., 2016; Bertram and Chi, 2018). Retailers make their mobile websites more user-friendly and provide more product alternatives, competitive prices, and a pleasant shopping experience to bring more income. Investment in mobile commerce does not guarantee consumer satisfaction or anticipated returns (Swilley, 2015). According to Park et al. (2015), consumer purchasing decisions are heavily influenced by brand image. It is also vital to note that the quality of an online retailer’s website affects the likelihood of a customer making a purchase.

Among the world’s fastest-growing economies, Pakistan is one of the fastest-growing globally. The mobile sector in Pakistan is one of the most developed globally. Pakistan has the potential to become one of the world’s most digitally integrated economies due to technological advancements in the business sector. Pakistan has the potential to become the smartphone-savvy nation of the world. There were approximately 125 million mobile users in Pakistan by year’s end of 2015. This revolutionized Pakistan’s business culture and procedures. Mobile technology has also changed the way businesses and customers attachment. The smartphone industry has overtaken the mobile sector and in 2015, smartphone accounted for about a third of the market, which is an entirely different situation currently (Imtiaz et al., 2015). According to a German market research agency, Pakistan has overtaken the United Kingdom and the United States as the sixth fastest-growing smartphone market (Sheikh et al., 2016). Based on population, Pakistan has a higher percentage of smartphone users than India. From 2015 to 2016, smartphone sales in Pakistan have grown by 780% (Imtiaz et al., 2015). The majority of Pakistan’s population is under 30 and uses technology often. Nearly 80% of internet users log on at least once a day. This population’s internet potential is significantly more significant. The portion of the population is being educated and influenced through social networks. For example, Facebook’s popularity has risen due to the ease with which it can now be accessed *via* cell phones and mobile phone numbers. According to Murphy et al. (2017), over 76% of Asians use Google’s mobile search engine. When the products are increased, it becomes a risk or issue for customers since they cannot choose a product as there are several distinct brands, prices, and product features (Talim et al., 2021). When the people want to make purchases and cannot believe that all the brands are not trustworthy, it is difficult for them to buy, and the brand or company product cannot grasp what they want (Demir et al., 2021).

Mobile commerce has played a significant role in social commerce. According to Murphy et al. (2017), about 3.3 billion smartphone users worldwide, this massive number of smartphone users strongly influences customer purchasing habits (Malik et al., 2021). As smartphone users are becoming more connected with other customers and become more affected by others, they have a massive effect on another life (Malik et al., 2021). The capacity of a purchaser’s intentions to specify their buying behavior by using the internet is known as online purchase intention (PI) (Leiss et al., 2013). Marketing now has a more significant impact on customers (Sangvikar et al., 2019). In the present era, purchasing decisions are made online and are impacted by the amount of information available. Customers are being bombarded with more information on the internet every day, which has substantially lowered their attention span (Veer et al., 2019). Many organizations have suffered financial difficulties in the market (Kolte et al., 2018; Haq et al., 2022). The typical customer evaluation cycle’s period has been dramatically lowered and turned into a new shape. Marketers must rethink their marketing communication tactics. The modern consumer’s decision-making process is quite fluid. It starts with recognizing a need, acquiring information, and finally, a dynamic evaluation of the options. Customers may now obtain any information in less time on a worldwide scale, thanks to the impact of the internet and m-commerce (Taylor et al., 2017).

Consumer PI prediction during offers can be defined as a binary classification. Customers are divided into two groups based on whether the customers want to purchase intents or not (Sismeiro and Bucklin, 2004; Ling et al., 2019). According to a literature review, the direct association between brand perceived value and PI is already studied (Chattalas and Shukla, 2015; Hennigs et al., 2015). Research has discovered a correlation between brand perceived value and consumer PI (Jung Choo et al., 2012; Chattalas and Shukla, 2015; Cheah et al., 2015; Hennigs et al., 2015). Consumers who believe a brand provides the value they assume and demand are willing to buy it again and again, suggest it to others, and pay a higher rate even though there are more good products available at a reasonable price by the competitors (Khan et al., 2021; Petraviit et al., 2021). As a result, subjective customer perceived expectations about premium brand value are essential for specific brands (Hennigs et al., 2015). According to studies, brand perceived value has a favorable impact on brand purchase intent (Hung et al., 2011; Cheah et al., 2015). According to a prior study, customer PI is influenced by perceived brand value, although the effect of this value varies among nations and cultures (Petraviit et al., 2021).

In the last 20 years, the business world has altered dramatically due to the growth of the mobile technologies and internet. The “digital transformation of marketing,” is represented in such a method that businesses and customers have adopted new technologies and, most interestingly, how technology has permitted innovative market behaviors, experiences, and interactions (Lamberton and Stephen, 2016). Technological innovations offer us new possibilities, allowing suppliers to “connect with consumers to co-create creative goods, services, and experiences” (Payne et al., 2008, p. 88). M-commerce, similar to the arrival of e-commerce, is having a significant impact on how companies and people engage with each other (Huang et al., 2015). Mobile phones have become indispensable in users’ daily lives, and they give an excellent framework for companies to connect, communicate with, and serve clients at any time and from any location (Wang et al., 2015). According to Mahapatra (2017), due to external reasons of ease, mobile devices have become excellent mediums for purchasing experiences (search, possession, evaluation, and post-purchase). Several requests have been made to understand service quality in the context of a rapidly developing smartphone industry, especially since users might estimate service quality aspects differently in online and mobile settings (Arcand et al., 2017). Firms must adopt mobile channels and build effective programs that improve and explain the benefits and value for customers in order to engage and sustain mobile customers (Laukkanen, 2016). Industry practitioners, and academic researchers are very keen to more interested in determining the potential of m-commerce (Sun and Chi, 2018; Rana et al., 2019). Because m-commerce is steadily becoming more inexpensive for customers throughout the world, the chances of firms, particularly SMEs, succeeding in the m-commerce industry are higher nowadays than in the past (Rana et al., 2019). It is observed that consumers have low purchasing intents regarding various products (Wang and Somogyi, 2018). Many companies have failed to develop consumer-friendly m-commerce (Cohen, 2013). Although the phenomenon of buying intent has been extensively studied, more research is required (Hoque et al., 2018). This research is carried out on consumers of m-commerce done in Pakistan and uses perceived influence, virtual interactivity, brand image, brand expected value, and PIs and finds the direct and indirect relationship between these variables. In the next section, this research describes the literature review, relationships of variables, and their hypothesis; in the next section, current research proposes the methodology; in the next chapter, recent research suggests the results and discussion, and in the last portion, this study presents Conclusion, Recommendations, Future Directions Research Implications of the study.

### Purchase Intentions

A customer’s propensity to buy a product or service is their PI. In other words, the buyer expects that they will purchase the product after it has been evaluated (Torlak et al., 2019). “Customer willingness to buy a product or service is PI” (Sanny et al., 2020). “An evaluation of the possibility that a buyer would buy a help or item” is commonly used to describe PI (Talim et al., 2021). An important consideration in determining whether or not to buy something is the quality of the product. In a perpetual cycle of improvement, the ongoing modifications to items increase their performance, and thus the customer’s expectations are met (Talim et al., 2021). Customers are more likely to purchase an item with higher quality; hence, quality should be enhanced every second (Talim et al., 2021). Customers’ PIs are positively influenced by product quality, according to the researchers’ findings. Examined the more significant quality item makes higher buy objectives toward lower-quality things (Mirabi et al., 2015). In addition to providing their customers with emotional and sensory benefits, brands are essential for building substantial brand value (Talim et al., 2021). Perceived credibility, trust, perceived risk, subjective norms, and social norms are some of the potential determinants that can be explained by the theory of planned behavior (Oputa and Bin Ahmad, 2019) argue that advertising plays a significant role in influencing Malaysian consumers’ perceptions of mobile devices.

### Virtual Interactivity

There are three basic types of interactions that might take place: face-to-face engagement between individuals, online contact between individuals, and interaction between individuals and machines (Domagk et al., 2010). The second kind of interaction is referred to as “virtual interactivity.” Virtual interactivity is also defined as “the extent to which internet users are involved in changing the content of sites in real-time,” according to Steuer (1992). Using Steuer’s (1992) definition of virtual interaction as a guide, this study will examine the three characteristics of virtual interaction: (1) two-way communication, (2) timely communications, and (3) mutual controls, all of which have been evaluated and intensely evaluated and supported by empirical research (Yoo et al., 2010; van Noort et al., 2012; Hu et al., 2016; Yoo and Park, 2016). Even though some researchers believe that businesses may overestimate the benefits of customer engagement (Kunkel et al., 2019), the majority of studies suggest that increased interaction with customers is beneficial in terms of raising customer satisfaction and increasing customer perceived value (Yoo et al., 2010; Vendemia, 2017). Besides that, customer happiness and perceived value are highly connected with brand awareness and brand image.

Additionally, Barreda et al. (2016) found that website interactivity had a beneficial impact on brand awareness and brand image in the hotel industry. Virtual interactivity, in our opinion, can be divided into two categories: interactive diversity and interactive frequency. One of the most important aspects of interaction intensity, according to Tajvidi et al. (2021), is frequency. When it comes to public management, regular communication is essential to clarify expectations, reduce the gap between the formulations of policies, and implement those policies. As a result of this, according to Barreda et al. (2016), the more information organizations have about their consumers and the information they are seeking, the more positively they will be perceived by their customers as a result of the information gathering effect. According to Wang and Emurian (2005), long-term trust is created through repeated encounters and long-term partnerships over time. The second point is that buyers may see platform and form diversity as two distinct degrees of interaction variety. In 2013, 77% of Fortune 500 firms were active on Twitter, 70% were active on Facebook, and 69% were active on YouTube; according to Barnes-Mauthe et al. (2013), brands have created a robust online presence through social media.

In another way, most of these businesses maintain many social media profiles. In addition to ensuring that potential followers are covered extensively, different platform linkages will also increase the influence of each platform. According to us, the variety of ways in which celebrity photographs are displayed online, particularly in informal settings, is critical to the success of online advertising. According to Colliander and Marder’s (2018) study, “in social media, photographs with a snapshot aesthetic elicit greater brand sentiments and intents to suggest people to follow the Instagram account” compared to those with traditional studio aesthetics, our hypothesis is supported. Online celebrities are encouraged to provide more informal and beautiful images and music and films to showcase their talents and creativity. Consumers’ awareness, emotion, and behavior are influenced by interactivity, which is increasingly relevant to internet flows (van Noort et al., 2012; Hussain et al., 2021). As a result, it is been found that engaging customers virtually can help them remember a brand and influence their impressions of it (Liu et al., 2020). It is argued that virtual interactivity positively influences brand image (Liu et al., 2020). Moreover, Barreda et al. (2016) examine that virtual interactivity positively impacts consumer PI.

Based on these facts, current research argues the following hypothesis.

**H2:** Virtual interactivity has a positive impact on consumer purchase intention.

**H4:** Virtual interactivity has a positive impact on brand image.

### Perceived Influence

When it comes to marketing their brands and products, companies are increasingly using social media to develop strong ties with their customers (Shiau et al., 2018). Researchers and practitioners interested in brand challenges and social media platforms have taken notice of this fact (Alalwan et al., 2017). Even though countless research has been conducted on the subject, only a few have examined how businesses might utilize social media to develop branding and marketing approaches (Ananda et al., 2016; Hudson et al., 2016).

When it comes to social media marketing, enlisting important influencers to sway potential customers is expected to improve interactions with customers, provide added value to those customers, boost the effectiveness of marketing efforts, and benefit the company (Ananda et al., 2016; Alvi et al., 2020). As a result of the rise of digital influencers, the relationship between businesses and their target audiences on social media platforms and online social networks has undergone a sea change. Media has opened to outsiders with a professional or hobbyist approach to social media production and promotion (e.g., blogging and creative activities), with a structured relationship with advertisers and an interconnectedness to their audience, as well as a desire to gain social visibility and prestige due to web-based technologies’ ability to approach the audience directly (Abidin, 2015, 2016, Duffy and Hund, 2015; Pedroni, 2016; Rocamora, 2018). It is common for firms to compensate digital influencers for their loyalty to their brands by providing them with free products, the opportunity to get “exposure,” a small quantity of money, or even the expectation that they will work for free to promote their products (Scott, 2015; Duffy, 2016; Rocamora, 2018). When it comes to brand-related information, digital influencers are seen as a chance to widen the scope of eWOM. Even though influencers are widely recognized as online opinion leaders because of their authenticity, expertise, and knowledge, few studies have been conducted from an opinion leader perspective on digital influencers (e.g., Du and Li, 2011; De Veirman et al., 2017; Magno, 2017; Casaló et al., 2020). Research based on experimental and qualitative data challenges the notion that increasing popularity might lead to perceptions of opinion leadership and influence followers’ brand attitudes and purchase behavior (De Veirman et al., 2017; Djafarova and Rushworth, 2017). Smith-Ditizio and Smith (2022) explain the concept of brand image based on media dependency theory. Brand image and PI are positively influenced by virtual interactivity (Liu et al., 2020).

A theory called observational learning theory (OLT) asserts that individuals acquire their attitudes and behaviors toward consumers due to their encounters with and learning from external socialization agents (Keaveney and Parthasarathy, 2001) such as friends, classmates, and social media. An essential part of the communication process, this interaction usually serves a social role in developing a connection with the interlocutor (Shen and Sengupta, 2018) and can ultimately lead to better engagement with the message content (Mollen and Wilson, 2010; Kapitan and Silvera, 2016; Mohd-Ramly and Omar, 2017). To make matters worse, when the message is coming from a group of people they look up to, people utilize the brand connected with that message to help them build an idea of who they are (Escalas and Bettman, 2003). Even though there is no direct interaction between the follower and the digital influencer, a psychological connection is established between the two that allows for identification with the message source and internalization of the message (Abidin, 2015; Kapitan and Silvera, 2016). These considerations lead us to believe that followers who digital opinion leaders influence display higher involvement with the recommended companies by incorporating them into their self-concept, thus contributing to building their identity (Escalas and Bettman, 2003; Sprott et al., 2009). Jiménez-Castillo and Sánchez-Fernández (2019) examined that perceived value has a positive effect on PIs (Liu et al., 2020). Based on these facts, current research argues the following hypothesis.

**H1:** Perceived influence has a positive impact on consumer purchase intentions.

**H3:** Perceived influence has a positive impact on brand image.

### Brand Expected Value

Expectations and perceived values can be shaped by other people’s opinions, decisions, and behaviors, according to a wide range of studies (Zeithaml, 1988; Zeithaml et al., 1993; Sridhar Balasubramanian, 2001; Weiss et al., 2008; Al-Debei and Al-Lozi, 2014). People’s ability to generate an opinion on the value of a product or service is influenced by various factors, including the amount of information they have access to (Kim and Han, 2009; Al-Debei and Al-Lozi, 2014). M-commerce may influence customer perception of the product value in an online context (Gruen et al., 2006). This suggests that the value expectations of their followers are shaped by the influence exerted by the digital influencers they endorse.

It is widely accepted in the academic literature that perceived value is a significant predictor of purchase intent (Cronin et al., 2000; Leroi-Werelds et al., 2014; Gallarza et al., 2017; Wang, et al., 2022). Using perceived value as an alternative to customer satisfaction as an indication of customer loyalty is trustworthy to a previous study (Mencarelli and Lombart, 2017). Online settings have also been used to study the value–PI link. According to a survey by Bonsón et al. (2015), perceived value is the most important antecedent of online PI, showing that the more consumers believe that an item on a travel website has a high perceived value; the more likely they are to buy from that website. As Wu et al. (2014) demonstrated empirically, as customers’ perception of value increases, their repurchase intention increases in online contexts. Because of this, it stands to reason that, in the context of digital influencer–follower relationships, those who have high-value expectations for a brand promoted by influencers is also more likely to buy that brand. Choosing digital influencers is a calculated risk since organizations want to ensure that the recommended brands’ target audiences will find, buy, and use the products recommended by the influencers they choose (Uzunoğlu and Kip, 2014).

As a result of the brand’s popularity, buyers are less likely to feel the impacts of risk when purchasing from well-known businesses (Sabir et al., 2013; Liao et al., 2016). Customers’ expectations of such brands are so high that they are more likely to purchase. As a result, the more well-known a brand is, the more potential customers are to confirm their purchase and make a second buy (Geraldine and Laurent, 2019). Jiménez-Castillo and Sánchez-Fernández (2019) examined that brand expected value has positive effect on consumer PIs. Based on these facts, current research argues the following hypothesis.

**H5:** Brand expected value has a positive impact on consumer purchase intentions.

### Brand Image

Customers’ subjective judgments and subsequent behaviors are influenced by brand image, which is an extrinsic cue when consumers evaluate a product/service before purchase (Zeithaml, 1988). As Ajzen and Fishbein (1975) put it: “Consumers examine the repercussions of alternative behaviors before engaging in them” (Yarimoglu and Binboga, 2019). The perceived value of the behavior and personal norms inform consumer behavior intention (Alvi et al., 2019; Yarimoglu and Binboga, 2019). As described by Keller (1993), a brand’s image is the perceptions consumers have about a brand as reflected by the brand connections they have in their minds. If a consumer has had a positive encounter with a brand or has been exposed to a brand’s messaging, the bond will be more robust (Alkhawaldeh et al., 2020). Characteristics, benefits, and attitudes are the three types of brand association that might differ in favorability, strength, and uniqueness (Zia et al., 2021). There are more positive feelings regarding a product’s features if the brand image is viewed positively (Hendrawan and Agustini, 2021). If you want to stand out from the competition, you need a strong brand image to help your customers identify your product or service (Huang et al., 2020). Ryu et al. (2019) examined that brand image has positively impact on brand expected value, and a similar result is observed by Lien et al. (2015).

**H6:** Brand image has a positive impact on consumer purchase intentions.

**H7:** Brand image has a positive impact on brand expected value.

Based on the above relationships current study proposes the following mediation hypotheses.

**H8:** Brand expected value mediating the relationship of perceived influence and consumer purchase intention.

**H9:** Brand image mediating the relationship between virtual interactivity and consumer purchase intention.

**H10:** Brand image mediating the relationship of virtual interactivity and brand expected value.

**H11:** Brand expected value mediating the relationship of brand image and consumer purchase intention.

### Hypothesized Research Model


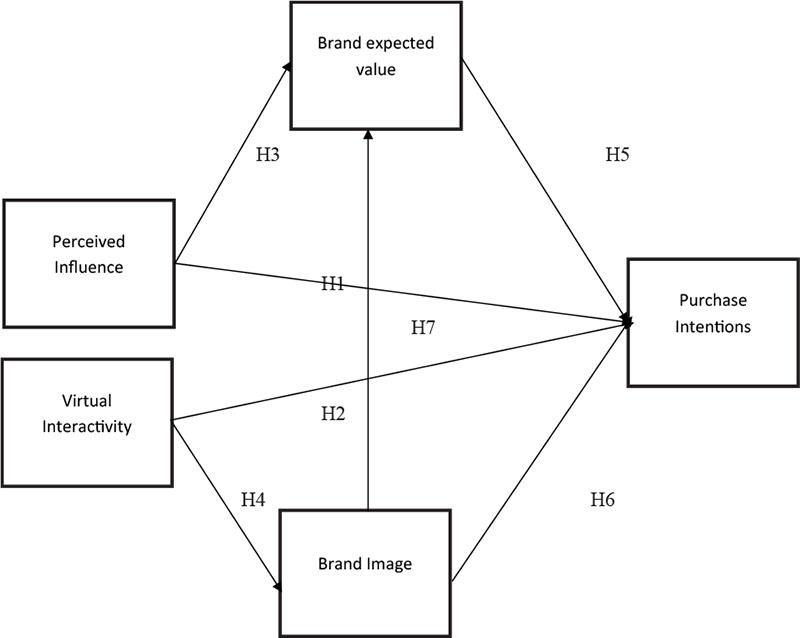


## Materials and Methods

### Target Population

Target population of current study are the consumers who are studying in Universities of Lahore and have android mobiles and engage in m-commerce.

### Sample Size

Data was collected from 227 customers who were engage in buying with the help of m-commerce.

### Sample Selection Procedure

Data collection is collected with the help of convenience sampling technique. To become the part of survey, first of all researchers ask the respondents about using the m-commerce for buying the products. If they answer is yes then they provide the questionnaire and request them to fill the questionnaire. Total 287 questionnaires were distributed. A total of 232 questionnaires were returned back, five questionnaires were incomplete and 227 complete questionnaires were used in final analysis.

### Scales and Measurements

For estimation of brand expected value, current study uses the 4-statements’ questionnaire of Jiménez-Castillo and Sánchez-Fernández (2019). Sample statement is “In my opinion, the products of the brands suggested by the influencers that I follow are well made.” Alpha of reliability is 0.91. Consumer PI, current study uses the 3-statements’ questionnaire of Jiménez-Castillo and Sánchez-Fernández (2019). Sample statement is “I would follow brand recommendations from the influencers that I follow.” Alpha of reliability is 0.92. For estimation of perceived influence, current study uses the 3-statements’ questionnaire of Walsh et al. (2014). Sample statement is “I value the opinion of the influencers that I follow as if they were someone close whom I trust.” Alpha of reliability is 0.95. For estimation of perceived influence, current study uses the 3-statements’ questionnaire of Walsh et al. (2014). Sample statement is “I value the opinion of the influencers that I follow as if they were someone close whom I trust.” Alpha of reliability is 0.81. Current study uses the 3-statements’ questionnaire of Jang et al. (2008) for estimating the virtual interactivity. Sample statement is “The information I exchange using consumer social network websites is useful for me.” Alpha of reliability is 0.95. Current study uses the 5-statements’ questionnaire of del Río et al. (2001) for estimating the brand image. Sample statement is “This brand is reliable.” Alpha of reliability is 0.93.

## Results

### Demographics

The demographic findings are summarized in Table 1. Male respondents participated in majority and accounted for about 62% of the total respondents compared to female respondents. In terms of age, nearly half of the respondents (50.66%) were between 21 and 30 years, followed by 41.41% who were between the ages of 31 and 40 years. The data also revealed that 5.73% of respondents were between the ages of 41 and 50, while only 2.20% were above 50 years and contributed the least to the study. According to the educational profile, more than half (i.e., 55.51%) of respondents held a bachelor’s degree, 22.47% had a master’s or higher degree, 14.54% held a higher secondary school certificate, and only 7.49% held secondary school certificate. The marital status of the respondents revealed that 60.79% were single, and 34.36% were married. While 2.20 and 2.64% were widowed and divorced, respectively. Lastly, income status revealed that the respondents having more than 50,000 rupees of income participated in the majority, followed by those with income between 41,000 and 50,000 rupees. The details of demographics are presented in Table 1.

**TABLE 1:** Demographics findings.

Criteria	Category	Frequency (*n* = 227)	Percentage
Gender	Male	140	61.67
	Female	87	38.33
Age	21–30 years	115	50.66
	31–40 years	94	41.41
	41–50 years	13	5.73
	51–60 years	5	2.20
Education	Secondary	17	7.49
	Higher secondary	33	14.54
	Bachelor	126	55.51
	Master and above	51	22.47
Marital status	Unmarried	138	60.79
	Married	78	34.36
	Widow	5	2.20
	Divorced	6	2.64
Income	Rs. 20K and below	15	6.61
	Rs. 21K–30K	26	11.45
	Rs. 31K–40K	17	7.49
	Rs. 41K–50K	48	21.15
	Above Rs. 50K	121	53.3

*$1 = Rs. 185; n, final responses for data analysis.*

### Common Method Bias

The data for this study were collected in a single sitting over 3 months and 24 days beginning in the first quarter of 2022. Podsakoff (2003) argued that CMV might arise when a researcher collects data in a single sitting. To circumvent this difficulty, both *a priori* and *post hoc* remedies were used. *Priori* remedies included an attachment of a cover letter, different rating scales, and questionnaire pre-testing, which helped to reduce informant effort in responding. As *post hoc* remedies, Harman single factor analysis and full co linearity test were used (Podsakoff, 2003). Harman’s single-component analysis revealed that the first factor accounted for just 43.248% of the total variance, less than 50% (Akter et al., 2017). Although the calculated total variance of the first factor was below 50%, it was close to the threshold. Therefore, the full collinearity test was also used to validate the Harman single factor analysis findings. The VIF values of all constructs were between 1.000 and 1.909 (≤3.3), indicating that CMV was not a significant problem in the data.

### PLS-SEM Analysis

The PLS-SEM analysis was applied using Smart PLS 3.3.3 (Ringle et al., 2015). Two stages of assessing the measurement model and evaluating the structural model were followed, as suggested by Hair et al. (2021). The reason of selection of PLS-SEM analysis is that model is complex that is why current study apply smart PLS.

### Measurement Model Assessment

The evaluation of Cronbach’s alpha, composite reliability (CR), factor loadings, AVE, and discriminant validity is part of the measurement model assessment stage. As shown in Table 2, the results reveal that the first four parameters met the threshold criteria (i.e., α > 0.7, CR > 0.7, AVE > 0.5, and loading > 0.7) set by Hair et al. (2021). It means that the model has sufficient reliability and convergent validity.

**TABLE 2:** Measurement model assessment: VIF, reliability, and convergent validity.

Construct	Item code	Loadings	VIF	α	CR	AVE
Brand image	BRAI1	0.836	2.147	0.890	0.919	0.695
	BRAI2	0.849	2.327			
	BRAI3	0.825	2.113			
	BRAI4	0.827	2.129			
	BRAI5	0.831	2.128			
Brand expected value	BREV1	0.861	2.228	0.872	0.913	0.723
	BREV2	0.847	2.110			
	BREV3	0.851	2.137			
	BREV4	0.842	2.030			
Perceived influence	PERI1	0.839	1.604	0.798	0.881	0.712
	PERI2	0.858	1.901			
	PERI3	0.834	1.695			
Purchase intentions	PURI1	0.883	2.148	0.852	0.91	0.772
	PURI2	0.852	1.894			
	PURI3	0.900	2.475			
Virtual interactivity	VIRI1	0.855	1.694	0.809	0.887	0.724
	VIRI2	0.836	1.733			
	VIRI3	0.861	1.936			

*VIF, variance inflation factor; α, Cronbach’s alpha; CR, composite reliability; AVE, average variance extracted.*

Next, discriminant validity was also investigated to ensure that each construct is fundamentally unique from the others. HTMT approach is deemed most robust than cross-loading and the Fornell–Larcker criterion to establish discriminant validity of the constructs (Hair et al., 2021). Table 3 revealed that the HTMT value of each construct was less than 0.85 threshold criteria, indicating that every construct of this study has appropriate discriminant validity. The VIF test was also applied to detect the collinearity issue before moving to structural model assessment. All values of VIF were found below 3; hence, no collinearity issue was detected in measurement models (Hair et al., 2021).

**TABLE 3:** Discriminant validity (HTMT <0.85).

Construct	Brand expected value	Brand image	Perceived influence	Purchase intentions	Virtual interactivity
Brand expected value					
Brand image	0.673				
Perceived influence	0.751	0.687			
Purchase intentions	0.771	0.691	0.703		
Virtual interactivity	0.170	0.262	0.140	0.232	

### Structural Model Assessment

The structural model assessment was evaluated once the measurement model was demonstrated to be reliable and valid. At this stage, coefficients of determination, prediction relevance, effect sizes, and hypotheses are tested.

### Coefficient of Determination and Predictive Relevance

First, coefficient of determination and predictive relevance were assessed. Table 4 summarizes its results. The *R*^2^ (0.476) explains that the brand image and perceived influence together contribute 46.6% brand’s expected value (Hair et al., 2021). Similarly, *R*^2^ (0.530) explains that all exogenous constructs such as virtual interactivity, perceived influence, brand image, and expected value contribute 53% of brand expected value. Whereas *R*^2^ (0.050) shows that brand image is only 5% explained by its exogenous constructs. Besides, Table 6 also indicates that all the values of *Q*^2^ are above zero; however, they fall in the weak to a substantial category of predictive relevance, which means the proposed model has weak significant predictive relevance (Hair et al., 2021).

**TABLE 4:** Coefficient of determination (*R*^2^) and predictive relevance (*Q*^2^).

Exogeneous construct	*R* ^2^	Decision	*Q* ^2^	Decision
Brand expected value	0.476	Substantial	0.339	Medium
Brand image	0.050	Weak	0.034	Weak
Purchase intentions	0.530	Substantial	0.400	Substantial

**TABLE 5:** Hypotheses testing: direct relationships.

Relationships	β	SD	*t*-Value	95% CI [LL, UL]	Decision	*f* ^2^
H1: Perceived influence → purchase intentions	0.178	0.064	2.762*	[0.076, 0.288]	Accept	0.036
H2: Virtual interactivity → purchase intentions	0.061	0.055	1.118*^NS^*	[−0.034, 0.152]	Reject	0.008
H3: Perceived influence → brand expected value	0.431	0.057	7.576*	[0.347, 0.523]	Accept	0.235
H4: Virtual interactivity → brand image	0.223	0.060	3.681*	[0.115, 0.321]	Accept	0.052
H5: Brand expected value → purchase intentions	0.398	0.058	6.905*	[0.291, 0.485]	Accept	0.177
H6: Brand image → purchase intentions	0.249	0.061	4.056*	[0.143, 0.348]	Accept	0.074
H7: Brand image → brand expected value	0.343	0.060	5.756*	[0.240, 0.434]	Accept	0.149

*p < 0.01; ^NS^not Significant. β, path coefficient; SD, standard deviation; CI, confidence interval; LL, lower limit; UU, upper limit.

**TABLE 6:** Hypotheses testing: indirect relationships.

Relationships	β	SD	*t*-Value	95% CI [LL, UL]	Decision
H8: Perceived influence → brand expected value → purchase intentions	0.172	0.033	5.257*	[0.122, 0.229]	Accept
H9: Virtual interactivity → brand image → purchase intentions	0.055	0.022	2.522*	[0.025, 0.097]	Accept
H10: Virtual interactivity → brand image → brand expected value	0.030	0.012	2.587*	[0.015, 0.054]	Accept
H11: Brand image → brand expected value → purchase intentions	0.137	0.033	4.108*	[0.088, 0.193]	Accept

*p < 0.01; ^NS^not significant. β, path coefficient; SD, standard deviation; CI, confidence interval; LL, lower limit; UU, upper limit. R^2^, coefficient of determination; Q^2^, predictive relevance.

### Hypotheses Testing (Direct Relationships)

**FIGURE 1:**
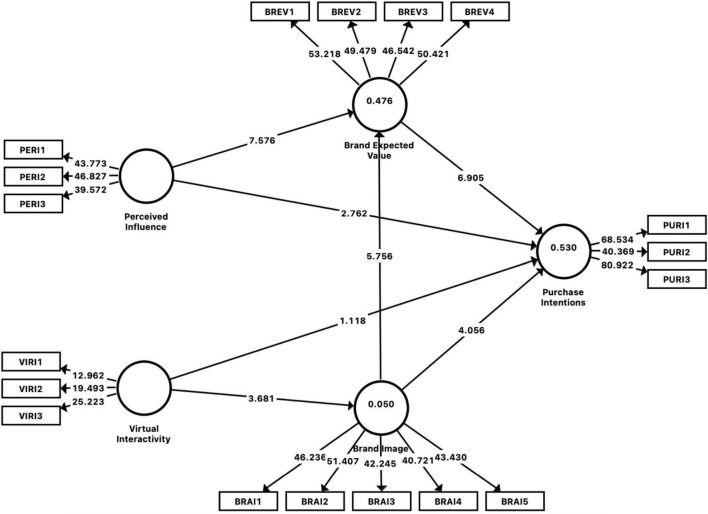
Structural model assessment.

Next, a bootstrap process with 5,000 iterations was run to test direct and indirect relationships, as shown in Figure 1 (Hair et al., 2021). The results of bootstrapping process are reported in Tables 4, 5. Table 4 revealed that all direct relationships except the relationship between virtual interactivity and PIs are positively significant.

Furthermore, Table 4 is also listed effect size (*f*^2^) values of every exogenous construct based on the PLS-SEM results. It shows that the exogenous constructs had a small to medium effect on the endogenous constructs involved in this study (Cohen, 2003).

### Hypotheses Testing (Indirect Relationships)

Similarly, Table 5 revealed that all indirect relationships are positively significant. For example, it showed that brand expected value positively and significantly mediate between perceived influence, brand image, and purchase intension. Similarly, brand image was also found to have positive and significant mediation between virtual interactivity, purchase intension, and brand expected value.

## Discussion

Results indicate that perceived influence has a positive impact on consumer PIs. The more well-known a brand is, the more likely customers are to confirm their purchase and make a second buy (Geraldine and Laurent, 2019). Jiménez-Castillo and Sánchez-Fernández (2019) observed that perceived influence positively impacts consumer PIs. This is also logical because customers of m-commerce and people are influenced by someone and engage in buying the product. Thus, H1 is accepted. The following result is the relationship between virtual interactivity and consumer PIs. Results show that virtual interactivity has an insignificant positive impact on consumer PIs because the beta value is insignificant. This result is that consumers of m-commerce are not much familiar with the virtual technology and virtual interactivity is not aware, so the result of this hypothesis is not significant. This result is also in line with previous research by Jiménez-Castillo and Sánchez-Fernández (2019). Thus, hypothesis no two is rejected.

Furthermore, perceived influence has a significant favorable impact on brand expected value. This result implies that consumers who are the critical consumers’ influence are more motivated to purchase the specific products and brands. The strength of this relation is 0.431. This result is also in line with previous research by Jiménez-Castillo and Sánchez-Fernández (2019). Thus, hypothesis no three is accepted.

Moreover, virtual interactivity has an insignificant positive impact on brand image. This result is similar to the argument of previous research, as a brand image is positively influenced by virtual interactivity (Liu et al., 2020). But the result of Liu et al., 2020 shows that virtual interactivity has an insignificant negative impact on brand image. Barreda et al. (2016) examine the significant positive effects of virtual interactivity on brand image. Results indicate that virtual interactivity positively impacts brand image, and the value of β is 0.223. Hence, hypothesis no four is accepted.

Additionally, brand expected value has a positive impact on consumer PI. This result is similar to the argument of previous research. As, Jiménez-Castillo and Sánchez-Fernández (2019) examined, brand expected value has a positive effect on consumer PIs. Results indicate that brand expected value positively affects consumer PIs, and the value of β is 0.398. Hence, hypothesis no five is accepted.

Additionally, brand image positively impacts brand expected value and consumer PI. This result is similar to the argument of previous research. Ryu et al. (2012) examined that brand image positively impacts brand expected value, and a similar result is observed by Lien et al. (2015). The brand image also positively affects brand expected value (Lien et al., 2015). Hence, hypotheses no. 6 and 7 are also accepted.

Results of mediation describe that all the mediational hypotheses are accepted. This means that brand expected value mediates the relationship between perceived influence and consumer PI, brand image mediates the relationship between virtual interactivity and consumer PI, brand expected value mediates the relationship between brand image and consumer PI, and finally, brand expected value mediating the relationship of brand image and consumer PI. These are all the findings of the current study. From the best of the researcher’s knowledge, no previous study finds the above-mentioned mediational relationships.

## Conclusion

The current study is designed to find the relationships between virtual interactivity, perceived influence, brand expected value, brand image, and consumer PI. The current study proposes the seven research hypotheses and four hypotheses for indirect relationships, i.e., mediations. For direct relationships, six hypotheses are accepted, while one is rejected (i.e., relationship of virtual interactivity with PIs). Perceived influence has a prominent impact on brand expected value. After that, brand expected value has a noteworthy impact on consumer PI. Similarly, four mediational hypotheses are accepted. Among all mediations, the brand expected value is a more effective partial mediator for the relation of perceived influence and PIs.

## Contribution of Research

The current study contributes in many ways. Firstly, in respect of consumer PI, current research adds to the body of literature by proposing a wider framework by including virtual interactivity, perceived influence, brand expected value, brand image, and consumer PI in a single model. Secondly, this research also adds to the body of literature by considering the consumers of m-commerce regarding the proposed model in the Pakistani cultural context. This research also generalizes the result of Barreda et al. (2016); they examine the significant positive impact of virtual interactivity on the brand image even though Liu et al., 2020 show that virtual interactivity has an insignificant negative impact on brand image. Thirdly, this research also proposes four mediation hypotheses. Fourthly, it is important to examine the impact of perceived influence on brand expected value and consumer PI because these relations are rarely examined in previous research (e.g., Magno, 2017; Casaló et al., 2020; Liu et al., 2020). Fifthly, it is widely believed that strong brands have a powerful impact on organizational success, and these brands can gain a competitive edge over other organization (Zhang et al., 2016). But results of the current study tell another story that perceived influence can also foster the consumer PIs, especially with the help of marketing the product *via* m-commerce. Sixthly, the framework current study can help organizations analyze consumer perceptions in changing environments, especially in marketing, with the help of m-commerce. Seventhly, this research also helps the managers that brand image and brand expected values are still helpful for increasing the consumer PI. So, organizations must formulate mixed policies with the help of a good brand for a brand less world.

## Limitation and Future Direction

This is cross-sectional research; thus, this research does not provide evidence of respondents’ responses to the proposed model. Hence, in future research, longitudinal data may help overcome this problem and will be beneficial for generalizing the results.

Similarly, most of the current study respondents belong young age fall between 21 and 40 years of age. So, it is beneficial for future research to focus on respondents of 41 to 60 years and compare their responses to respondents who fall in age interval 21 to 40 years. Additionally, most of the respondents of the current study are unmarried. In future research, similar research will be carried out on an equal sample of married and unmarried respondents and will compare the results of both samples. This research chooses the consumers of m-commerce; in the future, more respondents who use social media and purchase their products will consider for future research.

Some other variables that may influence consumer PI such as attitude toward the brand (Choi and Rifon, 2012; Haq et al., 2022), perceived influencer trustworthiness (Hsu et al., 2013; Magno and Cassia, 2018), level of involvement or influencer reputation (Hsu et al., 2013) should be investigated further in future research (Kapitan and Silvera, 2016). Future research could look into predictors of consumers’ perceptions of influence, like the lower-influencer emotional attachment (Moussa and Touzani, 2017), perceived quality (Wang and Lin, 2011), or online flow aspects (Lim, 2014). Due to time and cost constraints, the current study uses only one kind of virtual reality, i.e., virtual interactivity. In future research, more dimensions of virtual reality used by Yan et al. (2022), i.e., ease of use of VR, usefulness of VR, and authenticity of experience of VR-enabled services, will be used in future research.

## Data Availability Statement

The original contributions presented in this study are included in the article/supplementary material, further inquiries can be directed to the corresponding author.

## Ethics Statement

Ethical review and approval was not required for the study on human participants in accordance with the local legislation and institutional requirements. The patients/participants provided their written informed consent to participate in this study.

## Author Contributions

XJ helped in introduction write up and overall suggestions about improvement in manuscript. AA looked after overall the manuscript especially done the abstract, introduction, results and discussion, and conclusion portion. MN and NA did analysis portion. HZ helped in completing some portion of literature and some portion of introduction. All authors contributed to the article and approved the submitted version.

## Conflict of Interest

The authors declare that the research was conducted in the absence of any commercial or financial relationships that could be construed as a potential conflict of interest.

## Publisher’s Note

All claims expressed in this article are solely those of the authors and do not necessarily represent those of their affiliated organizations, or those of the publisher, the editors and the reviewers. Any product that may be evaluated in this article, or claim that may be made by its manufacturer, is not guaranteed or endorsed by the publisher.
